# Psychometric properties of brief-COPE inventory among nurses

**DOI:** 10.1186/s12912-021-00592-5

**Published:** 2021-05-06

**Authors:** Hanif Abdul Rahman, Wegdan Bani Issa, Lin Naing

**Affiliations:** 1 PAPRSB Institute of Health Sciences, Universiti Brunei Darussalam, Tungku Link Road, Gadong, BE1410 Brunei; 2 Research Institute for Medical and Health Sciences, Health Promotion Research Group, University of Sharjah, Sharjah, United Arab Emirates; 3 College of Health Sciences, Nursing Department, University of Sharjah, Sharjah, United Arab Emirates

**Keywords:** Brief-COPE, Coping, Nurses, Psychometrics, Reliability, Validity

## Abstract

**Objective:**

Brief-COPE inventory is widely used to assess coping; however, validation evidence is absent and previous results were inconsistent. This study aimed to validate psychometric properties of this inventory to ensure culturally appropriate usage.

**Methods:**

Preliminary validation study on 423 female nurses from the United Arab Emirates. Confirmatory factor analysis (CFA) with maximum likelihood estimation was computed to test four different previous models. Exploratory factor analysis (EFA) protocol was used to determine underlying domain structure of Brief-COPE in this population.

**Results:**

The four previous models on CFA had inadequate fit indexes. Two-factor (22-items) second-order model that explained 37.0% of the total variance with Cronbach’s alpha at 0.81 and 0.88, respectively, was suggested.

**Conclusion:**

This validation of Brief-COPE is timely considering nurses enduring different types of stressors. In addition, cultural diversity needs to be considered in coping research. Re-assessment of this exploratory structure is necessary to ensure accurate measurement of coping strategies.

## Highlights

- Nurses faces persistent demands and new challenges in modern healthcare
- Coping strategies are important mechanism to mitigate adverse effects of high stress environment that has personal and organisational implications
- Brief-COPE has been extensively used in nursing research, however, its psychometric properties need to be evaluated
- Cultural diversity has significant influence towards the constructs of brief-COPE inventory

## Introduction

Coping with persistent demands and challenges in the modern healthcare system is increasingly necessary among healthcare professionals [1]. Nurses, in particular, faces high stress and burnout levels from increased workload and work engagement, emotional exhaustion, staff shortages and poor health [2]. Studies have utilized different measurement tools to assess nurses’ coping strategies, in various settings [3, 4].

One of the most widely used measures of nurses’ coping strategies is the Brief-COPE (Coping Orientation to Problems Experienced) inventory, a shortened version of the full 60-items (16 scales) COPE inventory developed by Carver, Scheier, & Weintraub [5]. Based on the Folkman & Lazarus’ Ways of Coping model and the Behavioural self-regulation model, the inventory aimed to assess various positive and negative coping strategies effectively [5]. The inventory was further refined to reduce participant fatigue and item redundancies and eventually the creation of the Brief-COPE inventory, which consists of 28-items with 14 scales [5].

Following sound theoretical guidance, a number of health-relevant studies have provided empirical evidences by using statistical tools to extract underlying domains and test psychometric properties in various settings and populations such as caregivers of people with dementia in the United Kingdom [6], among breast cancer chemotherapy patients in Malaysia [7], people with traumatic brain injury in New Zealand [8], people living with HIV in China [9], person living with HIV/AIDS in India [10], pregnant minority women in the United States [11], adults in Italy [12], and community population in Chile [13]. Brief-COPE is also translated in a number of languages such as French version [14], Malay version [7], Brazilian-Portuguese version [15], and Chilean version [13].

Although several coping strategies instruments exists such as the Folkman and Lazarus’s Ways of Coping questionnaire [16], the Multidimensional Coping Inventory [17] and the Coping Inventory for stressful situation [18], the relatively lengthy items were not particularly useful in long research protocols and clinical research. The 28-items Brief-COPE do not have this shortcoming, and have been used in many studies extensively. However, the validation results have not always been consistent due to the complex nature of coping dimensions on different types of stressors and study populations. In addition, we have not found studies examining validity and reliability estimates of Brief-COPE on nurses in the United Arab Emirates (UAE). In the UAE, majority of nurses are expatriates and coming from diverse cultural backgrounds, predominately Pilipino, Indian, Pakistani, and people from Western and other Arab countries [19]. As nurses move from their own home countries to a different culture of the UAE, they face different types of stressors related to reallocation which require them to develop new coping mechanisms in response to new emerging stressors. The newly stress coping strategies; however, must consider Islamic values and modesty standards of the UAE culture to enhance their acceptance by colleagues and healthcare organization [20]. Therefore, understanding stress coping strategies of nurses, who comprise the largest segment of healthcare system, will guide healthcare policy formation geared toward enhancing positive stress coping mechanisms of nurses to facilitate their smooth transition, minimize their turn over and enhance their job satisfaction. Therefore, this paper aimed to bridge this gap by validating the psychometric properties of Brief-COPE inventory to ensure culturally appropriate usage.

## Materials and methods

### Study design and setting

This preliminary validation study investigated the psychometric properties of the English version of Brief-COPE inventory among nurses working in the United Arab Emirates (UAE). Participants were recruited between October 2017 and December 2018 using cluster sampling.

### Eligible population

Eligible participants were female nurses aged ≥20 years who were not pregnant. We excluded men in the current study because the number of male nurses is very minimal in the country. Participants who had chronic health conditions (e.g., diabetes mellitus, hypertension, coronary artery disease, renal disease) and those taking oral contraceptive pills were excluded from this study. The exclusion is guided from previous studies where coping mechanisms for these group of population is significantly different from general population [21].

### Data collection procedure

Trained research assistants visited the selected clusters to recruit participants. Advertisements about the study including an invitation to participate were sent to the selected sites before the site visits. The research assistants then met nurses, screened interested nurses for eligibility, introduced the study, obtained consent, and asked participants to complete the questionnaire.

### Research instrument

The original brief-COPE by Carver [5] comprised of two-items in each 14 subscales including self-distraction, active coping, denial, substance use, use of emotional support, use of instrumental support, behavioral disengagement, venting, positive reframing, planning, humor, acceptance, religion, and self-blame. Meyer [22] categorized these subscales into second-order factor model, which consisted of “Adaptive coping strategies” (use of emotional support, positive reframing, acceptance, religion, humor, active coping,, planning, and use of instrumental support) and “Maladaptive coping strategies” (venting, denial, substance use, behavioural disengagement, self-distraction, and self-blame). Cooper, Katona, Orrell, & Livingston [23] further categorized the original subscales into three, which consisted of “Emotion-focused strategies” (use of emotional support, positive reframing, acceptance, religion, and humor), “Problem-focused strategies” (active coping, planning, and use of instrumental support) and “Dysfunctional coping strategies” (venting, denial, substance use, behavioural disengagement, self-distraction, and self-blame). More recently, Eisenberg, Shen, Schwarz, & Mallon [24] indicated two major, i.e., “Approach coping” (active coping, emotional support, use of instrumental support, positive reframing, planning, and acceptance) and “Avoidant coping” (self-distraction, denial, substance use, behavioural disengagement, venting, and self-blame) that excludes humor and religion.

### Statistical analyses

Brief-COPE inventory has 4-point Likert response from 1 (I haven’t been doing this at all) to 4 (I’ve been doing this a lot). Mean and standard deviation of the item scores and observed range as well as floor and ceiling effects where more than 15% is considered significant effect [25], were calculated. To evaluate the construct validity of the brief-COPE inventory, a series of confirmatory factor analysis (CFA) with maximum likelihood estimation was computed to test four different previous models including the original structure by Carver [5], and other studies by Meyer [22], Cooper et al. [23], and Eisenberg et al. [24] that are labelled as Model 1, Model 2, Model 3, and Model 4 respectively. Various model fit indices were considered: 1) relative chi-square (χ2), 2) χ2/df where less than 2 is good fit, 3) root mean square error of approximation (RMSEA) where ≤0.08 is considered good fit, 4) Non-normed fit index (NFI) where ≥0.95 is considered good fit, 5) Comparative fit index (CFI) where ≥0.90 is considered good fit, 6) Goodness-of-fit index (GFI) where ≥0.95 is considered good fit, and 7) Akaike’s information criterion (AIC) where lower values is considered better model [26].

The four models had inadequate fit indexes; thus, we performed exploratory factor analysis (EFA) model using maximum likelihood technique with varimax rotation to determine number of factors extracted and ascertained with scree plot, eigenvalue value more than 1, and factor loadings of more than 0.4. Prior to this, KMO value (≥ 0.5) and Bartlett’s sphericity test (*p <* 0.001) were computed [27]. Finally, a structural equation diagram with standardized regression estimates was rendered to visualize the EFA model. Item-total correlation coefficients and Cronbach’s alpha coefficients where ≥0.70 is considered good internal consistency [28] were also estimated. All analysis was computed using R 3.6.3 [29] and RStudio for Mac [30].

### Ethical considerations

This study was approved by [masked]. All procedures followed ethical standards in accordance to the Helsinki Declaration of 1964 and later versions. Written informed consent were obtained from participants prior to study commencement, and kept secure in a locked cabinet by the research team.

## Results

423 valid data from nurses in UAE who completed the brief-COPE questionnaire, were included in this analysis. Participants had mean age of 36.7 years (*SD* 8.5 years). Majority was expatriates (85.1%) from different nationalities and 68.4% were married.

Table 1 summarize the results of CFA model fit indices with respect to Model 1 to Model 4. Chi-square test of absolute model fit (*p <* 0.001) and other indices including NFT, CFI and GFI indicated all models had poor absolute fit. RMSEA index indicated only Model 1 had cut-off ≤0.08, a good fit. AIC considered Model 4 was comparatively a better model than the others.

**Table 1:** CFA Model Fit Indices of Brief-COPE on previously specified models (*n* = 423)

	Scales	Dimension	Items	χ2	χ2/ df	RMSEA	NFI	CFI	GFI	AIC
Model 1	14	0	28	940.0	3.63	0.079	0.802	0.845	0.866	26,950
Model 2	14	2	28	1485.7	4.48	0.091	0.688	0.737	0.793	27,350
Model 3	14	3	28	1511.8	4.54	0.091	0.682	0.731	0.791	27,374
Model 4	12	2	24	1197.6	5.01	0.097	0.679	0.722	0.805	23,507

Model 1 (Carver, 1997) Model 3 [28]

Model 2 [29] Model 4 [30]

χ2(df) (Chi-square (degree of freedom)). *RMSEA* Root Mean Square of approximation, *NFI* Non-normed fit index, *SRMR* Standardized Root Mean Square Residual, *CFI* Comparative fit index, *GFI* Goodness of fit index, *AGFI* Adjusted Goodness of fit index, *AIC* Akaike information criterion

Based on the original 14 subscales of brief-COPE inventory, CFA resulted on highly cross-loaded estimates on all factors except for behavioural disengagement, self-blame, substance use, humor, religion, and acceptance. Emotional support and use of informational support loaded on the same factor. Likewise, positive reframing and planning also formed a single factor.

**Fig. 1:**
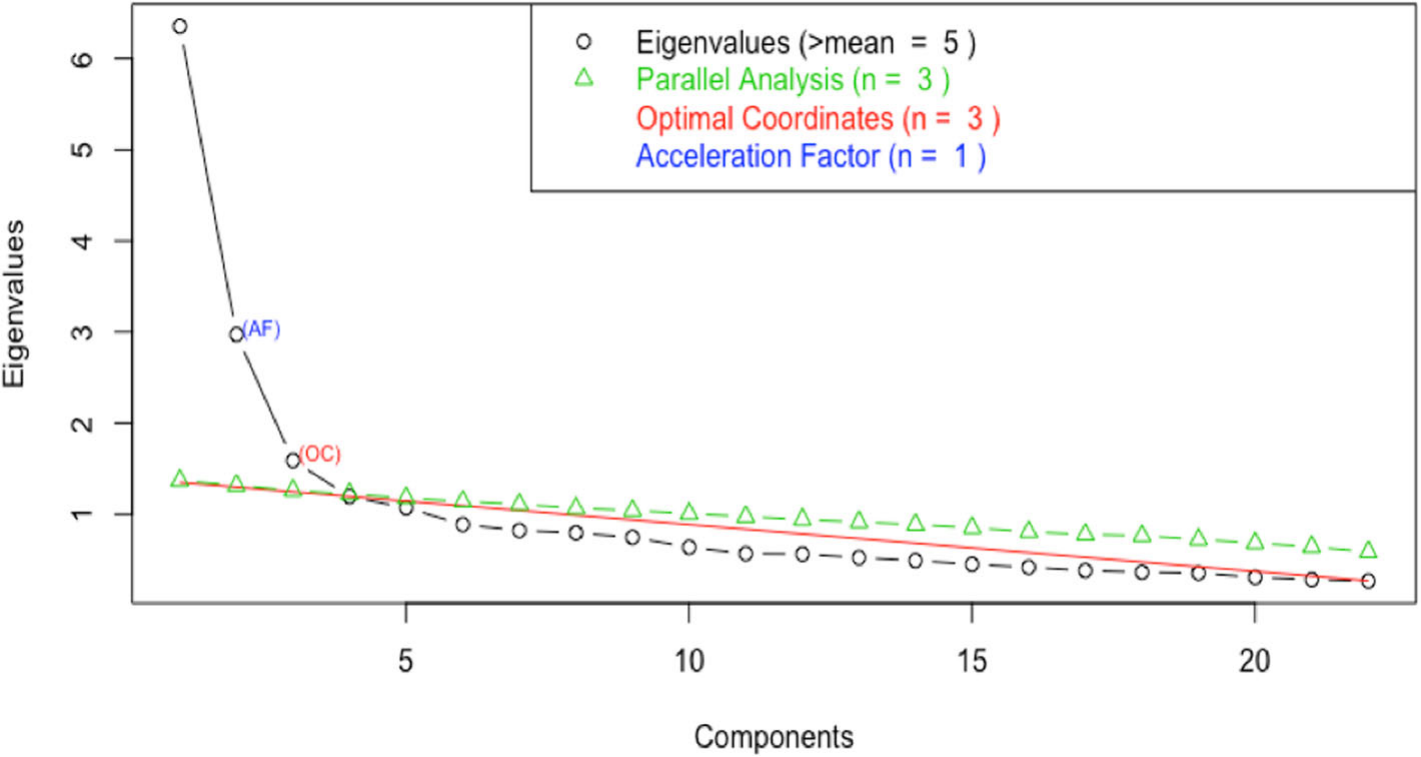
Scree plot of Exploratory Factor Analysis with Eigenvalues and Optimal factor selection (*n* = 423)

Due to poor model fit with of CFA models and high cross-loadings, EFA was conducted to establish factor structure for our study population. The KMO value (0.85) and Bartlett’s sphericity test (χ2 = 4632.7, df = 378, *p <* 0.001) indicated good factorability. The scree plot and eigenvalue more than 1 suggested seven factors with five-point optimal extraction (Fig. 1). Varimax rotation with factor loadings more than 0.4 yielded two factor extraction (11 scales excluded humor, self-distraction, and substance use), derives EFA model accounting for 37% of the total variance with the following fit indices: χ2/df = 4.74, RMSEA = 0.099, NFI = 0.75, GFI = 0.84, AIC = 21,790.8. Item-total correlation coefficients ranged from 0.37 to 0.69 and 0.52 to 0.63 for Factor 1 and Factor 2, respectively. Table 2 details the loadings and reliability estimate for this EFA model as well as mean and standard deviation of the scores and floor and ceiling effects.

**Table 2:** Mean scores, Distribution, Factor loadings and Reliability estimates of Brief-COPE (*n* = 423)

	Scales (Range 2–8)	Item no.	Mean score (SD)	Floor (%)	Ceiling (%)	Factor Loadings	Alpha (scales)	Alpha (factor)
**Factor 1**	Self-distraction	1	5.1 (1.5)	5.0	5.0	–	–	0.81
		19				–		
	Denial	3	3.7 (1.5)	28.8	1.9	0.45	0.56	
		8				0.63		
	Substance use	4	2.4 (0.9)	81.3	0.2	–	–	
		11				–		
	Behavioral disengagement	6	4.2 (1.7)	19.1	5.4	0.58	0.68	
		16				0.65		
	Venting	9	4.7 (1.5)	6.6	5.0	0.53	0.45	
		21				0.31		
	Self-blame	13	4.5 (1.7)	14.4	5.7	0.73	0.75	
		26				0.73		
	Humor	18	4.5 (1.8)	19.6	5.9	–	–	
		28				–		
**Factor 2**	Active coping	2	5.8 (1.4)	1.4	11.1	0.48	0.53	0.88
		7				0.62		
	Emotional support	5	5.3 (1.6)	2.4	13.0	0.52	0.67	
		15				0.61		
	Information support	10	5.3 (1.6)	3.5	12.5	0.54	0.61	
		23				0.65		
	Positive reframing	12	5.6 (1.5)	2.1	13.2	0.49	0.54	
		17				0.65		
	Planning	14	5.7 (1.5)	3.5	12.3	0.51	0.60	
		25				0.53		
	Acceptance	20	5.7 (1.5)	2.8	16.3	0.64	0.65	
		24				0.58		
	Religion	22	6.4 (1.6)	1.7	35.7	0.61	0.79	
		27				0.57		

Exploratory factor analysis (Varimax). *SD* Standard deviation *Alpha* Cronbach’s alpha

**Fig. 2:**
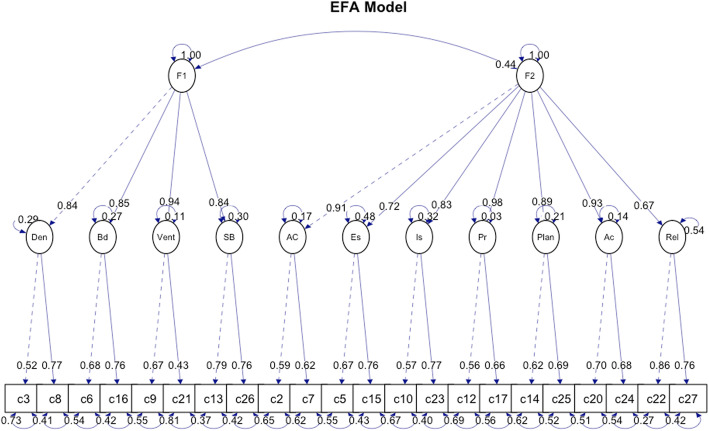
Structural EFA model of Brief-COPE inventory (*n* = 423)

Initial analysis showed skewed distributions of the responses on several items. Floor effects were present for denial, substance use, and behavioural disengagement. Ceiling effects were significant for acceptance subscale. Although Cronbach’s alpha coefficient for first-order subscales were mostly below 0.70 except for self-blame and religion, there was good alpha estimate for the second-order two-factor EFA model at 0.81 and 0.86, respectively, where the structure is illustrated in Fig. 2.

## Discussion

This study has established the psychometric properties of the Brief-COPE inventory among nurses working in the UAE. Similar to previous studies, our validation estimates have demonstrated inconsistencies with previous models due to the complex nature of coping especially in different parts of the world that could be influenced by culture, different generations, types of stressors, and study populations. This study is timely considering that COVID-19 pandemic has affected health professionals worldwide. In UAE, a total of 250,000 cases have been recorded with 740 deaths, as of 20 December 2020 [31]. Nurses, as front liners, is even more pressing to ensure instruments measuring their coping strategies are both valid and reliable [32–34].

A number of studies has used Brief-COPE to examine nurses coping strategies [35–37], however, in the absence of validation study, the results reported may be problematic and disputed. In particular, the CFA models using structure from previous studies did not achieve good fit, observed high cross-loadings, and Cronbach’s alpha of individual subscales were inadequate (although most were above 0.5), which was similarly reported in previous validation studies including the original Brief-COPE study [9, 12, 13, 38]. Nevertheless, the developer of Brief-COPE reported that the flexibility of the inventory encourage researchers to apply and test on different settings and population to extensively either using all the subscales or selectively use relevant scales in their research [38].

Even though Brief-COPE was developed with sound theoretical model, existing validation studies of different version of brief-COPE have produced large variation in empirical evidences, particularly on the dimensionality of the scales, which may limit comparability of results in studies using this inventory. Mohanraj et al. [10] exploration of the Tamil version of Brief-COPE underlying structure among person living with HIV/AIDS in India, yielded a five factor (17-items) model accounting 41.5% of the total variance. Yusoff et al. [7] investigation on Malay version of Brief-COPE among adolescent in Malaysia, produced a nine factor model explaining 71.2% of the total variance. Su et al. [9] generated a six factor model on the Chinese version of Brief-COPE, which explained 55.5% of total variance. Reich, Costa-Ball, & Remor [39] constructed a four factor (24-items) model of the Uruguayan version, and more recently, Gloria & Peña [40] produced an eight-factor (24-items) model on the Chilean version. In our study examining nurses in the UAE, we suggested a two-factor (22-items) model that explained 37.0% of the total variance. Substance use subscale was excluded because UAE is an Islamic country, and items on alcohol is not relevant. Self-distraction and Humor subscales were slightly difficult to justify, however, considering highest score was Religion subscale, we could postulate that religiosity would most likely have preceded them.

### Limitations

We expected that the reduced items will compromise theoretical content of the inventory and future researcher should attempt to re-establish content validity with area experts and replicate the results of our study, and if possible, to conduct full validation study incorporating estimates for criterion validity and test-retest reliability.

## Conclusion

Overall, a two-factor second-order model provides a good model to interpret coping strategies among nurses in the UAE, which is timely considering nurses around the world is currently battling different types of stressors in addition to the COVID-19 pandemic situation. Our study also highlighted the need to account for cultural diversity in coping research as studies on translated version of Brief-COPE had yielded inconsistent underlying constructs. Re-assessment of this exploratory structure is critically warranted on nurses from different study settings and population to ensure accurate measurement of coping strategies.

## Data Availability

The datasets generated and/or analysed during the current study are not publicly available due to institutional data sharing clause but are available from the corresponding author on reasonable request.

## References

[1] Robertson HD, Elliott AM, Burton C, Iversen L, Murchie P, Porteous T (2016). Resilience of primary healthcare professionals: a systematic review. Br J Gen Pract.

[2] Yu F, Raphael D, Mackay L, Smith M, King A (2019). Personal and work-related factors associated with nurse resilience: a systematic review. Int J Nurs Stud.

[3] Isa KQ, Ibrahim MA, Abdul-Manan H-H, Mohd-Salleh Z-AH, Abdul-Mumin KH, Rahman HA (2019). Strategies used to cope with stress by emergency and critical care nurses. Br J Nurs.

[4] Lee HF, Kuo CC, Chien TW, Wang YR (2016). A meta-analysis of the effects of coping strategies on reducing nurse burnout. Appl Nurs Res.

[5] Carver C, Scheier M, Weintraub J (1989). Assessing coping strategies: a theoretically based approach, journal of personality and social psychology. J Pers Soc Psychol.

[6] Cooper C, Katona C, Livingston G. Validity and reliability of the brief COPE in Carers of people with dementia: the LASER-AD study. J Nerv Ment Dis. 2008;196 https://journals.lww.com/jonmd/Fulltext/2008/11000/Validity_and_Reliability_of_the_Brief_COPE_in.7.aspx, 11, 838, 843, DOI: 10.1097/NMD.0b013e31818b504c. 10.1097/NMD.0b013e31818b504c 19008735

[7] Yusoff N, Low WY, Yip CH. Cope Scale : a Study on Malaysian Women Treated With: MPJ Online Early; 2009. p. 1–9.

[8] Snell DL, Siegert RJ, Hay-Smith EJC, Surgenor LJ (2011). Factor structure of the brief COPE in people with mild traumatic brain injury. J Head Trauma Rehabil.

[9] Su X-Y, JTF L, WWS M, Choi KC, Jian FT, Chen X (2015). A preliminary validation of the Brief COPE instrument for assessing coping strategies among people living with HIV in China. Infect Dis Poverty.

[10] Mohanraj R, Jeyaseelan V, Kumar S, Mani T, Rao D, Murray KR, Manhart LE (2015). Cultural adaptation of the brief COPE for persons living with HIV/AIDS in southern India. AIDS Behav.

[11] Ruiz RJ, Gennaro S, O’Connor C, Marti CN, Lulloff A, Keshinover T, Gibeau A, Melnyk B (2015). Measuring coping in pregnant minority women. West J Nurs Res.

[12] Monzani D, Steca P, Greco A, D’Addario M, Cappelletti E, Pancani L (2015). The situational version of the brief COPE: dimensionality and relationships with goal-related variables. Eur J Psychol.

[13] García FE, Barraza-Peña CG, Wlodarczyk A, Alvear-Carrasco M, Reyes-Reyes A. Psychometric properties of the brief-COPE for the evaluation of coping strategies in the Chilean population. Psicol Reflex e Crit. 2018;31(22):1-11. 10.1186/s41155-018-0102-3 PMC6967273 32026069

[14] Muller L, Spitz E (2003). Multidimensional assessment of coping: validation of the brief COPE among French population. Encephale..

[15] Brasileiro SV, Orsini MRCA, Cavalcante JA, Bartholomeu D, Montiel JM, Costa PSS (2016). Controversies regarding the psychometric properties of the Brief COPE: The case of the Brazilian-Portuguese version “COPE Breve.”. PLoS One.

[16] Folkman S, Lazarus RS (1988). Coping as a mediator of emotion. J Pers Soc Psychol.

[17] Endler NS, Parker JD (1990). Multidimensional assessment of coping: a critical evaluation. J Pers Soc Psychol.

[18] Endler NS, Kantor L, Parker JDA (1994). State-trait coping, state-trait anxiety and academic performance. Pers Individ Dif.

[19] Bani-Issa W, Radwan H, Al Marzooq F, Al Awar S, Al-Shujairi AM, Samsudin AR (2020). Salivary cortisol, subjective stress and quality of sleep among female healthcare professionals. J Multidiscip Healthc.

[20] UAE Government website. About the UAE. 2021. https://u.ae/en/about-the-uae/culture. Accessed 3 Apr 2021.

[21] Villada C, Hidalgo V, Almela M, Mastorci F, Sgoifo A, Salvador A (2014). Coping with an acute psychosocial challenge: behavioral and physiological responses in young women. PLoS One.

[22] Meyer B (2001). Coping with severe mental illness: relations of the brief COPE with symptoms, functioning, and well-being. J Psychopathol Behav Assess.

[23] Cooper C, Katona C, Orrell M, Livingston G (2006). Coping strategies and anxiety in caregivers of people with Alzheimer’s disease: the LASER-AD study. J Affect Disord.

[24] Eisenberg SA, Shen B-J, Schwarz ER, Mallon S (2012). Avoidant coping moderates the association between anxiety and patient-rated physical functioning in heart failure patients. J Behav Med.

[25] Terwee CB, Bot SDM, de Boer MR, van der Windt DAWM, Knol DL, Dekker J, Bouter LM, de Vet HCW (2007). Quality criteria were proposed for measurement properties of health status questionnaires. J Clin Epidemiol.

[26] Hu L, Bentler PM (1999). Cutoff criteria for fit indexes in covariance structure analysis: conventional criteria versus new alternatives. Struct Equ Model A Multidiscip J.

[27] Dziuban CD, Shirkey EC (1974). When is a correlation matrix appropriate for factor analysis? Some decision rules. Psychol Bull.

[28] Streinmer DL (1993). A checklist for evaluating the usefulness of rating scales. Can J Psychiatr.

[29] R Core Team (2021). R: A language and environment for statistical computing.

[30] RStudio Team (2020). RStudio: integrated development for R.

[31] Worldometers. COVID-19 Coronavirus Pandemic. 2020. https://www.worldometers.info/coronavirus/. Accessed 28 Apr 2020.

[32] Siyu C, Xia M, Wen W, Cui L, Yang W, Liu S, et al. Mental health status and coping strategy of medical workers in China during The COVID-19 outbreak. medRxiv. 2020;:2020.02.23.20026872. 10.1101/2020.02.23.20026872.

[33] Huang L, Ming XF, Rong LH. Emotional responses and coping strategies of nurses and nursing college students during COVID-19 outbreak. medRxiv. 2020;2020.03.05.20031898. 10.1101/2020.03.05.20031898.

[34] Zhuang Z, Zhao S, Lin Q, Cao P, Lou Y, Yang L, et al. Preliminary estimation of the novel coronavirus disease (COVID-19) cases in Iran: a modelling analysis based on overseas cases and air travel data. Int J Infect Dis. 2020;94:29-31. 10.1016/j.ijid.2020.03.019 PMC7194910 32171951

[35] McMeekin DE, Hickman RL, Douglas SL, Kelley CG (2017). Stress and coping of critical care nurses after unsuccessful cardiopulmonary resuscitation. Am J Crit Care.

[36] Alharbi H, Alshehry A (2019). Perceived stress and coping strategies among ICU nurses in government tertiary hospitals in Saudi Arabia: a cross-sectional study. Ann Saudi Med.

[37] Fathi A, Simamora RH. Investigating nurses’ coping strategies in their workplace as an indicator of quality of nurses’ life in Indonesia: a preliminary study. In: IOP conference series: Earth and Environmental science: IOP Publishing; 2019. p. 12031.

[38] Charles S (1997). Carver. You want to measure coping but your protocol’s too long: consider the brief COPE. Int J Behav Med.

[39] Reich M, Costa-Ball CD, Remor E (2016). Psychometric properties of the brief COPE in a sample of Uruguayan women. Av en Psicol Latinoam.

[40] Gloria C, Peña B (2018). Propiedades psicométricas del Cuestionario Brief Cope 28 en población Chilena expuesta a eventos altamente estresantes. XVI Coloquio Panamericano de Investigación en Enfermería.

